# Identification and Simultaneous Determination of the Main Toxical Pyrrolizidine Alkaloids in a Compound Prescription of Traditional Chinese Medicine: Qianbai Biyan Tablet

**DOI:** 10.1155/2021/5209618

**Published:** 2021-09-09

**Authors:** Mengting Yang, Daopeng Tan, Anjing Lu, Lin Qin, Changhong Wang, Hua Ling, Yanliu Lu, Yuqi He

**Affiliations:** ^1^Key Laboratory of Basic Pharmacology of Ministry of Education and Joint International Research Laboratory of Ethnomedicine of Ministry of Education, School of Pharmacy, Zunyi Medical University, Zunyi, Guizhou 563000, China; ^2^State Key Laboratory of Functions and Applications of Medicinal Plants, Guizhou Medical University, Guiyang 550014, China; ^3^The Institute of Traditional Chinese Medicine, Shanghai University of Traditional Chinese Medicine, Cailun Road 1200, Shanghai, China; ^4^School of Pharmacy, Philadelphia College of Osteopathic Medicine, 625 Old Peachtree Rd NW, Suwanee, GA 30024, USA

## Abstract

Qianbai biyan tablet (QT) is a compound prescription of traditional Chinese medicine which is used to treat nasal congestion, rhinitis, and nasosinusitis, with *Senecio scandens* as its main plant material. Several pyrrolizidine alkaloids (PAs) were reported in *Senecio scandens* and others of *Senecio* species. Although *Senecio scandens* is assigned as the legal plant material of QT, whether replaced use of it by other *Senecio* plants can bring toxicity is unknown because of the lack of quantitative data about toxic PAs between different *Senecio* species. In the present study, adonifoline, senkirkine, and another PA presumed as emiline have been identified in QT; however, there was no senecionine detected in all tablets. PA contents in QTs varied in different companies and different batches. Adonifoline existed only in *Senecio scandens*, and senecionine was detected in all eight *Senecio* plants investigated in the present study. Data showed that replaced use of *Senecio scandens* with a low level of senecionine by other *Senecio* plants such as *Senecio vulgaris* containing a high level of senecionine is advertised to be forbidden. Data of the present study may be used as a reference to make new drug quality regularity and recommendation guideline for the safety of QT.

## 1. Introduction

Qianbai biyan tablet (QT), a compound prescription of traditional Chinese medicine (TCM) used in China, South East Asia, and some European countries, was used to treat several clinical diseases such as nasal congestion, rhinitis, and nasosinusitis for several decades and had a large number of consumers. According to the China Pharmacopoeia (CP), *Senecio scandens* Buch.-Ham. ex D. Don was used as the main component of QT mixed with six other herbs, including Herba Selaginellae, Rhizoma Seu Radix Notopterygii, Semen Cassiae, Herba Ephedrae, Rhizoma Chuanxiong, and Radix Angelicae Dahuricae [1]. Some active constituents in QT, such as ephedrine from Herba Ephedrae, chrysophanol from Semen Cassiae, and flavonoids from Herba Selaginellae, were assayed to evaluate the quality of this tablet [2, 3]. To our knowledge, however, there were hardly any reports about the determination of toxic constituents in QT.

Pyrrolizidine alkaloids (PAs) were a kind of compounds that come from esterification of carboxylic acid named necic acid and pyrrole ring named necine and considered as one of the most toxical constituents in *Senecio* plants, causing hepatotoxicity [4] with the major clinical symptom of venoocclusion disease (VOD) and other toxicities including neurotoxicity and gene toxicity [5]. The mechanism of the toxicity of PAs was considered as metabolic activation [6] by liver P450s. In the world, about 3% of all flowering plants and more than 6000 plant species contain PAs [7], such as Compositeae, Boraginaceae, and Liguminosae [8]. Among these plants, *Senecio* plants (Compositeae) were considered the primary source of PAs, and large numbers of reports attributed the PAs toxicity against livestock, wildlife, and humans to *Senecio* plants [9].

In our previews [8], several PAs, including senecionine, senecionine N-oxide, seneciphylline, seneciphylline N-oxide, and senkirkine, existed in *Senecio scandens* that were the main components of QT as mentioned above [1]. The last one was an OTO-type PA, and others were RET-type; all of these five compounds were considered as toxicants because of their unsaturated C1,2-bond, which was considered as the key reason leading PAs induced toxicity [10]. Among these five compounds, senecionine was the common constituent existing in several other *Senecio* plants, such as *Senecio ambraceu*s [11], *Senecio* vulgaris [12], and *Senecio scandens* [13]. Senkirkine was the only otonecine-type PA found in *Senecio scandens*. Another probable toxical RET compound named adonifoline, which had unsaturated C1,2-bond and existed in *Senecio scandens*, was also reported, and the data showed that plants came from the different place had various content of adonifoline [14]. However, there was hardly any report about the toxicity of adonifoline, including acute toxicity and cumulative toxicity. PAs that existed in *Senecio scandens* may cause potent dangers for the consumption of herbs, even compound prescriptions. However, the types and quantities of PAs existing in QT and the content of PAs in QT were still unknown.

Although *Senecio scandens* is the active pharmaceutical ingredient of QT, other *Senecio* species such as *Senecio nemorensis*, *Senecio vulgaris,* and *Senecio cannabifolius* distribute extensively in Europe, China, and other Asian countries may be misused confused as a medical material. Tens of PAs, such as senecionine [12], senecicannabine [15], jacozine [16], seneciphylline [17], senecionine N-oxide [12], adonifoline [18] and senkirkine [19] have been found in *Senecio* plants. However, to our knowledge, except *Senecio scandens* there was rare information about the quantitative determination of the main PAs in these plants and whether the replacement of *Senecio scandens* by other *Senecio* plants in QT could bring toxicity or increase toxicity is unknown.

In the present study, we performed an LC/MS/MS analysis to identify the PAs in QT from different manufactories and different batches of the same manufactory, as well as related herbs of *Senecio* species described above. Several PAs such as adonifoline and senkirkine, which represented RET-type PAs and OTO-type PAs, respectively, have been detected in QT. Senecionine, which is the most common PA and had demonstrated toxicity, was assayed in all tablets and *Senecio* plants investigated in the present study. The purpose of this study was to provide the type and content of PAs in QT and give a toxicity evaluation to QT based on the chemical analysis data and other published work; on the other hand, whether replaced use of *Senecio scandens* by others could be promised was discussed here. Data of the present study can provide a reference for the quality standard of QT in later drug regulations.

## 2. Experimental

### 2.1. Chemicals

Reference substances including adonifoline, senecionine, senkirkine, monocrotaline, isoline, and clivorine were isolated by our lab from various *Compositae* plants such as *Senecio scandens* and *Ligularia duciformis*, and the purities of reference substances are more than 98%, determined by HPLC-DAD.

*Senecio* plants investigated were collected by our lab. All samples were authenticated by Dr. Lihong Wu, and voucher specimens were deposited in the herbarium of Shanghai R&D Center for Standardization of TCM. Samples of QT were purchased from drug stores all over China. Different batches of QT were obtained from Qixing Pharmaceutical Company Limited (Guangdong province, China). Detailed information about plants and tablets is shown in Table 1.

HPLC-grade acetonitrile was purchased from Sigma-Aldrich (St. Louis, MO, USA). HPLC-grade formic acid was purchased from Tedia Company (Fairfield, OH, USA). HPLC-grade water was produced by a Milli-Q water purifying system. All other chemicals and agents were purchased from commercial sources and were of the highest purity grade commercially available.

### 2.2. Instruments

The analysis was performed using a Thermo Finigan HPLC instrument equipped with a quaternary pump, a PDA detector, an autosampler, and a column compartment. The HPLC system was coupled with an ion trap mass spectrometry of LCQ XP Plus via an ElectroSpray Ionization (ESI) interface for mass analysis and detection. Data was acquired by the Xcalibur software obtained from Thermo Finigan. A Heraeus Biofuge Primo (Germany) was used for centrifugation in the present study.

### 2.3. Sample Preparation

Ten tablets of each sample were ground to a fine powder (40 mesh) using a pulverizer, an aliquot of 300 mg powder was accurately weighted to a 25 mL sealed conical flask, and 10 mL HCl solution (0.2%) was added to soak the powder for 30 min and then extract it by ultrasound for 40 min. Transfer the ultrasound extracted solution to a 15 mL centrifuge tube to perform centrifugation at 5000 × g for 10 min; the supernatant was filtered with a 0.3 *µ*M filter; an aliquot of 10 *µ*L was injected into LC/MS for identification and determination of PAs. The plant of *Senecio scandens* and other plant materials of *Senecio* species were also ground to the powder of 40 mesh, an aliquot of 1 g was accurately weighted to the 25 mL sealed conical flask, and the later operation was the same as tablets.

### 2.4. HPLC Analysis

Adonifoline, monocrotaline, isoline, clivorine, and senkirkine were chosen to optimize the chromatographic separation parameters. Chromatographic separation was performed on the Phenomenex Synersi MAX-RP C12 column (4 um, 250 × 4.6 mm) together with a supported C12 guard column. The mobile phase consisted of 1% formic acid in water as solvent A and acetonitrile as solvent B. The eluting process was as follows: beginning from 5% B and with a gradient from 5% to 28% B for 25 min, held at 28% B for 30 min, then to 95% B for 10 min, held at this percentage for 10 min. The mobile phase flow rate was 1 mL/min; the column temperature was set to 25°C, there was no use of the PDA detector because the UV absorption of pyrrolizidine alkaloids was very weak.

### 2.5. Mass Parameters

All mass spectrometric experiments were performed on an ion trap mass spectrometer. The LC effluent was introduced into the ESI source in a postcolumn splitting ratio of 10 : 1. The MS detector was optimized to obtain the maximum signal of [M+H]^+^ ions of isoline, which was one of the retronecine-type pyrrolizidine alkaloids, and clivorine, which was one of the OTO pyrrolizidine alkaloids. Scan mode was positive. The optimal parameters were as follows: Capillary Temp was 300.00°C, sheath gas flow and aux/sweep gas flow were 32 and 10, respectively (arbitrary unite), source voltage was 5 kV, source current was 80 uA, and capillary voltage was 9 V.

### 2.6. Identification of Main Pyrrolizidine Alkaloids in QT

A full scan mode was used to obtain the total ion chromatogram (TIC) of extracted tablet samples, and then the extracted ion chromatogram (EIC) was used to improve the signal-noise ratio (S/N ratio); based on TIC and EIC, several ions were screened as a candidate of probable PAs. [M + H]^+^ ions of even m/z number were chosen to perform MS/MS fragmentation after screening. The fragmentation regularity of PAs reported before in [20] was used to judge the type of candidate PAs based on the characteristic ions such as 120 and 138, which were used as the markers of RET PAs, as well as 122, 150, and 168, which were used as the markers of otonecine-type PAs. The fingerprint of MS/MS spectrum, as well as the retention retain time (RT) of probable peaks, was compared with that of the reference substances available.

### 2.7. LC/MS/MS Analysis of *Senecio* Plants and Pure PAs

The procedure described above was conducted on tablets to identify the probable PAs in QT while on eight *Senecio* plants, including *Senecio scandens, Senecio argunensis, Senecio cannabifolius* var. *integrifolius, Senecio cannabifolius, Senecio vulgaris, Tephroseris phaeantha, Senecio laetus, and Senecio nemorensis* to examine whether the plant source of QT contains these main PAs. Pure reference substance solutions such as adonifoline, senecionine, and senkirkine were injected to obtain their retain time, molecular weight, and MS/MS spectrum. Both the data of herbs and reference substance were used to compare with the main PAs in QT.

### 2.8. Validation of Analysis Method

#### 2.8.1. Standard Curve of Adonifoline, Senecionine, and Senkirkine

Reference substance solutions with a series of concentrations of pure adonifoline, senecionine, and senkirkine were injected to LC/MS/MS to obtain the SIC peak area or height for construction of the standard curve. The peak area of adonifoline in SIC 338 was linear with concentration range from 9.80 ng/mL to 6144.00 ng/mL; senkirkine in SIC 168 was linear with a concentration range from 10.00 ng/mL to 200.00 ng/mL. The peak area and height of senecionine in SIC 308 were linear, with a concentration range from 1.00 ng/mL to 5000.00 ng/mL.

#### 2.8.2. Precision and Accuracy

Reference substance of adonifoline was used to perform the valuation of analysis method, including precision and accuracy. The standard curve of adonifoline was analyzed for triplication in one day and in three consecutive days to calculate the interday and the intraday precision. 10 mL solution with concentrations of 1228.80, 245.80, and 49.20 ng/mL of adonifoline dissolved by 0.2% hydrochloric acid solution was added to the three aliquots of 300 mg powder of tablets (Rensheng, Guangxi Province, China), and preparations were conducted as described in Sample Preparation. The recovery of adonifoline was assayed to represent the accuracy of the analysis method; 10 mL 0.2% hydrochloric acid solution was added to another aliquot of 300 mg powder of the same manufactory as the blank contrast sample.

### 2.9. Assay of Four PAs in Eight *Senecio* Plants and QTs from Different Companies and Batches

**Figure 1:**
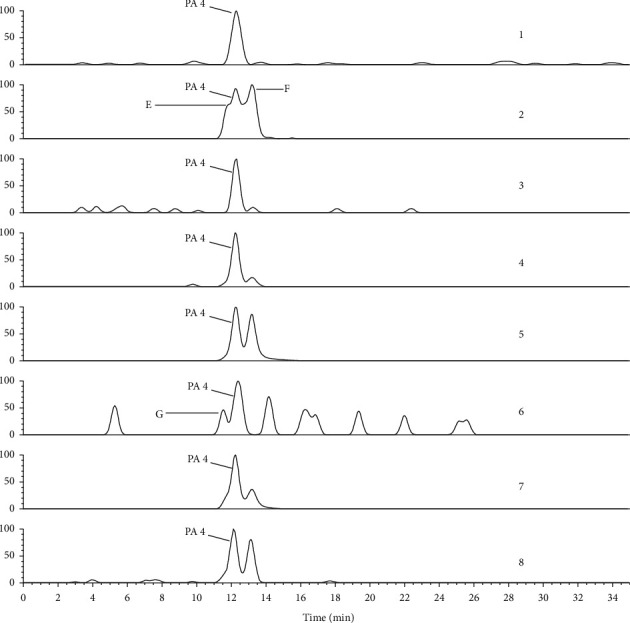
Chromatogram of total MS/MS fragment ions of 336 of eight *Senecio* plants. According to identification, PA4 is senecionine and E, F, and G are three other PAs which are the isomers of senecionine. Plant materials are as follows: 1*, Senecio scandens;* 2, *Senecio argunensis*; 3, *Senecio cannabifolius* var*. integrifolius*; 4, *Senecio cannabifoliu*s; 5, *Senecio vulgaris*; 6, *Tephroseris phaeantha*; 6, *Senecio laetus*; and 8, *Senecio nemorensis.*

QTs collected from 9 pharmaceutical companies, named Qixing (Guangdong province, China), Heping (Guangdong province, China), Guoyitang (Guangdong province, China), Huizhou (Guangdong province, China), Boluoxianfeng (Guangdong province, China), Baiyunshan (Guangzhou city, China), Rensheng (Guangxi province, China), Xiuzheng (Jilin province, China), and Sifang (Henan province, China), as well as 10 different batches from the same company (Qixing pharmaceutical company, Guangdong province, China) were assayed by LC/MS/MS. Selective ion chromatograms (SIC) of 338 which is the MS/MS fragment of the [M + H]^+^ ion 366, 308 which is the MS/MS fragment of [M + H]^+^ ion 336, and 168 which is the MS/MS fragment of [M + H]^+^ ion 366 were used to determine the content of adonifoline, senecionine, and senkirkine, respectively. Eight *Senecio* plants also were assayed the same as the tablets. Peak area was used for calculation of concentrations of adonifoline, senkirkine, and PA2, but peak height was used for senecionine because good separation of senecionine was not obtained in some plant samples (Figure 1). An external reference method was used in the assay of both tablets and plants.

## 3. Results

### 3.1. Chromatogram and MS/MS Fragmentation Regularity of PA Standard

Adonifoline, monocrotaline, isoline, senecionine, senkirkine, and clivorine were used to optimize chromatogram parameters. Because PAs possess the property of a weak base, a C12 column was chosen to prevent the tailing peak. 1% formic acid was used to keep the molecular state of PAs to improve the peak symmetry in the separation process and improve the ionization efficiency of PAs in the electrospray ionization (ESI) source. Because low concentrations were used in mixture standard, these compounds could not be observed in TIC but could be observed clearly in EIC of MS/MS fragment of their [M + H]^+^ ions.

**Figure 2:**
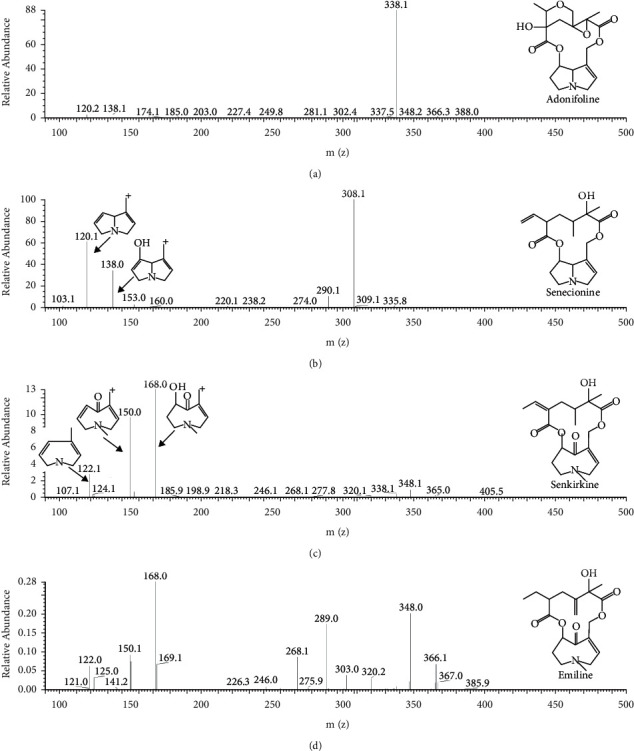
MS/MS spectrum of adonifoline (a), senecionine (b), and senkirkine (c) which were pure standard and PA2 (d) which was presumed as emiline.

MS/MS fragmentation of PAs was studied extensively [8, 20]. In the present study, the experiment was repeated on senecionine (RET) and senkirkine (OTO), and the same results as reported data have been reached. Ions 120 and 138 were usually generated by RET-type PAs, and 122, 150, and 168 were usually generated by OTO-type PAs under the positive scan mode. All these ions could represent the core structure of PA. Detail fragmentation regularities on the basis of senecionine and senkirkine are shown in Figures 2(b) and 2(c).

### 3.2. Identification of Pyrrolizidine Alkaloids in QT

**Figure 3:**
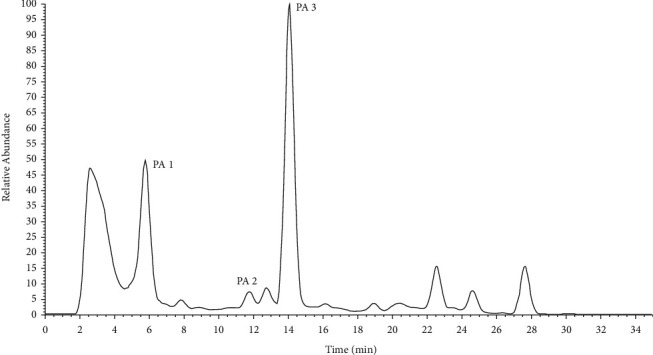
Chromatogram of total MS/MS fragment ion of 366 of QT produced by Baiyunshan Company. Except PA1 (RT 5.79 min), PA2 (RT 11.81 min), and PA3 (RT 14.10 min), other peaks in the chromatogram are non-PAs constituents.

**Figure 4:**
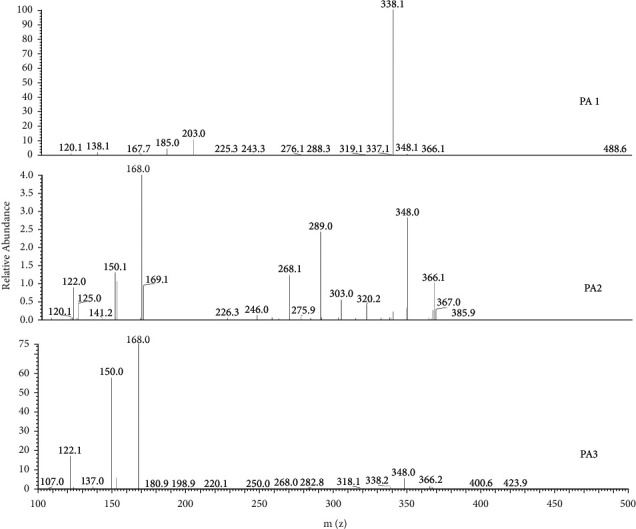
MS/MS spectrum of PA1, PA2, and PA3.

The identification of PAs in the QT from different manufactories was performed by mass fragmentation analysis and comparing with the reference standards. Firstly, full scan mode was used to obtain the molecular weight of probable PAs. After screening, three compounds, which had the same molecular weight of 365 and RT of 5.79 min, 11.81 min, and 14.10 min, were chosen as candidates of PAs labeled as PA1, PA2, and PA3 (Figure 3). Some samples investigated in the present study had one or two constituents of PA1, PA2, and PA3. Some samples contained all of the three candidates, such as tablets of Baiyunshan Company. Next, the [M + H]^+^ ions of candidates were used to perform the MS/MS fragmentation, and full scan mode was used to obtain the MS/MS spectrum of corresponded [M + H]^+^ ions. In the MS/MS spectrum of PA1, 120 and 138, which represent the RET PAs, were observed, while in that of PA2 and PA3, 122, 150, and 168, which represent the OTO PAs, were observed (Figure 4). Reported PAs, which had a molecular weight of 365, include three RETs, such as adonifoline [18], grantianine [21], and seneciocannabine [15], and two OTO such as senkirkine [19] and emiline [22]. It was reported that adonifoline and senkirkine existed in *Senecio scandens* which was the legal plant source of QT according to the CP, so these two compounds could be concluded as the most probable PAs in tablets. Except adonifoline and senkirkine, the last probable PA was of OTO, so emiline was the most probable candidate. Senecionine was the common constituent in most *Senecio* plants, but we did not detect the [M + H]^+^ ion 336 of senecionine in all tablet samples. Further, to verify the candidates of PAs in QT, their MS/MS fragmentation and retention time were compared to 6 PAs compounds, including monocrotaline, adonifoline, senecionine, senkirkine, isoline, and clivorine. Their retention time was 4.19, 5.79, 12.36, 14.10, 17.09, and 27.10 min, respectively. In the MS/MS spectrum of adonifoline, monocrotaline, senecionine, and isoline, two characteristic ions, 120 and 138, of RET PAs were observed; in that senkirkine and clivorine, 122, 150, and 168 were observed as the characteristic ions of OTO PAs. To identify the structure of PA1, PA2, and PA3, both RT and MS/MS spectrum of six references were used to compare with the candidates. As a result, PA1 was identified as adonifoline, and PA3 was identified as senkirkine. This corresponded with our conclusion about their structure. But PA2 was not the same as any one of the six PAs, and the most probable structure was presumed as emiline. Senecionine eluted at 12.36 min; although most *Senecio* plants, including *Senecio scandens*, contained this compound, there was no peak which possessed protonation molecular ion of 336 found in all QT samples. Structures and MS/MS spectrums of pure adonifoline, senecionine, and senkirkine are shown in Figures 2(a)–2(c), and probable structure and MS/MS spectrum of PA2 are shown in Figure 2(d).

### 3.3. Identification of Pyrrolizidine Alkaloids in *Senecio* Plants

**Figure 5:**
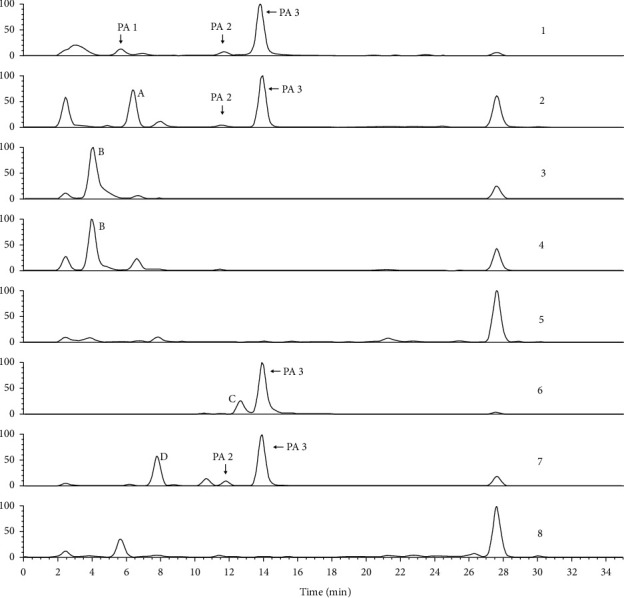
Chromatogram of total MS/MS fragment ion of 366 of eight *Senecio* plants: 1*, Senecio scandens;* 2, *Senecio argunensis*; 3, *Senecio cannabifolius* var*. integrifolius*; 4, *Senecio cannabifoliu*s; 5, *Senecio vulgaris*; 6, *Tephroseris phaeantha*; 6, *Senecio laetus*; and 8, *Senecio nemorensis.*

**Figure 6:**
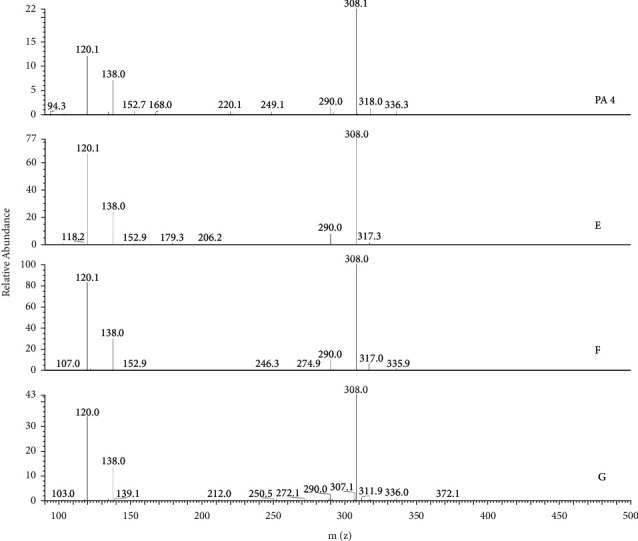
MS/MS spectrum of PA4 and peaks E, F, and G.

To confirm the PAs source in QT, eight *Senecio* plants, including *Senecio scandens, Senecio argunensis, Senecio cannabifolius* var. *integrifolius, Senecio cannabifolius, Senecio vulgaris, Tephroseris phaeantha, Senecio laetus,* and *Senecio nemorensis,* which are distributed extensively in China, were analyzed by LC/MS/MS in the present study. To identify PA1, PA2, and PA3 that were found in QT, a full scan of the MS/MS fragment of ions 366 was performed in all plants samples. In the TIC of MS/MS fragment of 366 (Figure 5), all of PA1, PA2, and PA3 were detected in *Senecio scandens*. Both of PA2 and PA3, but not PA1, were detected in *Senecio argunensis* and *Senecio laetus*. PA3 but not PA1 and PA2 was detected in *Tephroseris phaeantha,* which used to be *Senecio* plants. In *Senecio cannabifolius* var. *integrifolius*, *Senecio cannabifolius, Senecio vulgaris,* and *Senecio nemorensis,* all of PA1, PA2, and PA3 were not detected. Peaks A, B, C, and D are the other four PAs detected in *Senecio argunensis*, *Senecio cannabifolius* var. *integrifolius*, *Senecio cannabifolius, Tephroseris phaeantha,* and *Senecio laetus,* but not detected in QT. Other peaks in Figure 4 are not PAs. For confirming whether senecionine, which was reported in most *Senecio* plants and considered as the most common toxical PA in *Senecio* plants, existed in plant source of QT, the full scan of MS/MS fragment of 336, which is the [M + H]^+^ ion of senecionine, was performed to eight plant samples. In chromatogram of total MS/MS fragment of 336 (Figure 1), PA4 was found at RT 12.24 min. The MS/MS spectrum (Figure 6) indicated that it is senecionine by comparing with the standard substance. It is demonstrated by an LC/MS/MS method, for the first time, that all plant samples tested in the present study contain senecionine. In TIC of MS/MS fragment 336, the other three RETs were also detected and labeled as E, F, and and G; because of very similar MS/MS spectrum (Figure 6) and the same molecular weight with PA4, we conclude them as three isomers of senecionine. The detailed structures of E, F, and G need advanced investigation.

### 3.4. Validation of the Analysis Method

#### 3.4.1. Standard Curve of Adonifoline, Senecionine, and Senkirkine

The peak area of adonifoline in SIC 338 was linear with concentration range from 9.80 ng/mL to 6144.00 ng/mL, and that of senkirkine in SIC 168 was linear with concentration range from 10.00 ng/mL to 200.00 ng/mL. The peak area and height of senecionine in SIC 308 were linear with concentration range from 1.00 ng/mL to 5000.00 ng/mL.

#### 3.4.2. Precision and Accuracy

Average recovery of analysis method was 99.54% at high concentration, 96.31% at medium concentration, and 76.39% at low concentration, which indicated that accuracy in high and medium concentration is better than low concentration and some low content of PAs may be relatively inaccurate. Precision was 98.11% at high concentration, 97.20% at medium concentration, and 95.42% at low concentration for interday, while it was 88.29%, 85.20%, and 82.70% for intraday at high, medium, and low concentration, respectively.

### 3.5. Determination of Adonifoline, Senkirkine, Senecionine, and Emiline in QT and Eight *Senecio* Plants

Three compounds were identified as PAs in QT: two of them (PA1 and PA3) were sure to be adonifoline and senkirkine and PA2 was presumed as emiline. In eight *Senecio* species, PA1, PA2, and PA3 were found in different plants, and PA4 was detected and identified as senecionine in all eight *Senecio* plants. These four constituents in QT of 9 different pharmaceutical companies and in eight *Senecio* plants were assayed. The peak in SIM chromatogram was used for quantification. For adonifoline and senecionine, the selective ions were 338 and 308, which were the base peak in the MS/MS spectrum of their [M + H]^+^ ions, respectively. Ion 168, which was the characteristic ion of OTO PAs and the base peak in MS/MS spectrums of [M + H]^+^ ions of PA2 and senkirkine, was chosen to be the selective ion for PA2 and senkirkine. Peak area was used to determine the content of adonifoline, senkirkine, and PA2; but peak height was used to determine the content of senecionine because a good separation was not obtained for it (Figure 1). Because of no reference compound available, PA2 was semidetermined using pure senkirkine with one point external standard method. According to the determination result of four PAs in QT tablets of 9 companies and eight *Senecio* plants, senecionine was detected in all *Senecio* plants but not in any tablets. Adonifoline was detected as the main PA in most tablets but only in one *Senecio* plant (*Senecio scandens*). For understanding whether adonifoline contents in QT tablets coming from the same manufactory were the same, QT tablets of 14 batches from Qixing (Guangdong province, China) company were assayed in the present study.

According to the results of PAs content in QT tablets, the content of four PAs, including adonifoline, senecionine, senkirkine, and PA2, was significantly different in QT tablets from individual companies. Adonifoline was the most common constituent in QT, although no adonifoline was detected in Heping Co. and Rensheng Co., tablet of Qixing Co. has the highest content of adonifoline. Senecioine, the most common constituent in most *Senecio* plants which had noticeable toxicity, was not detected in any tablets. Except for Heping, Xiuzheng, and Sifang Co., senkirkine, an OTO PA, was contained in tablets of all other companies. PA2, which is presumed as emiline in the present study, was detected in tablets of Qixing, Guoyitang, Baiyunshan, and Rensheng Co. However, the quantities are very low. No PAs were detected in tablets of Heping Co., and the only adonifoline existed in tablets of Xiuzheng and Sifang Co. The results of PAs determination in tablets of different companies are expressed in Table 2.

According to the determination of PAs in tablets of different companies, adonifoline is the main constituent in QT. For understanding whether the PAs contents in the tablets of the same company were coincident, 14 batches of QT from Qixing Co. were collected to determine the content of adonifoline. As a result, only six batches contain adonifoline and the contents were various. All determination results are listed in Table 2.

*Senecio scandens* is the legal plant source of QT according to the CP, but in the actual production process, some other *Senecio* species may be used to replace it. In the present study, eight *Senecio* plants, including ones extensively distributed in China, were assayed to determine the content of adonifoline, senecionine, senkirkine, and PA2. As a result, *Senecio scandens* contained all four PAs, *Senecio argunensis* and *Senecio laetus* contained senecionine, senkirkine, and PA2 but no adonifoline, and *Tephroseris phaeantha* contained two PAs of senecionine and senkirkine. It was an exciting discovery that senecionine was detected in all plants tested in the present study, and adonifoline, the main constituent in QT, was detected only in *Senecio scandens*. All determination results are listed in Table 2.

## 4. Discussion

Three PAs (PA1, PA2, and PA3) were detected in QT. PA1 and PA3 were identified as adonifoline and senkirkine. PA2 was presumed as emiline in the present study. According to the determination result, adonifoline was the main RET PA and senkirkine was the main OTO PA in QT tablets. All three compounds had the unsaturated bond between C1 and C2 sites. According to the data reported, although this kind of structure is the key reason causing the PAs induced toxicity, the toxicity of adonifoline has not been reported. And, experiment data of our lab showed that the LD_50_ value of adonifoline on mice is far lower than senecionine (data not show). Other toxicities of QT induced by adonifoline should be investigated later by pharmacology method. Chemical analysis data cannot be used as the sole evidence to evaluate the toxicity of tablets but is an important part. Senkirkine was demonstrated as a toxical PA with mutagenic activity [23, 24]. The highest contents of senkirkine in QTs observed from samples investigated were several hundred nanograms per gram tablet and far lower than adonifoline. PA2 existed in tablets of Qixing, Guoyitang, Rensheng, and Baiyunshan Co., but the quantities were so small that it nearly cannot be detected.

Tablets of different companies were assayed in the present study. Data showed that PAs content in tablets of different companies was various. Fourteen batches of tablets of Qixing Co. were chosen to be assayed, and adonifoline, which was the main constituent in QT, was assigned as the index. The result showed that the contents of adonifoline in different batches of tablets were various. Contents of some batches were high, reaching more than 10 µg/g, but some batches contained no adonifoline. The factors that influence PAs content in QT may be concluded as the pharmaceutical technology, instability of PAs compounds, and the plant source of QT. In our opinion, plant source was proposed to be the main factor that makes the PAs content vary between different batches. Zhang et al. reported that adonifoline in *Senecio scandens* from a different province, even the same province of China, had various content [14]. Adonifoline content in *Senecio scandens* of Wuhu (Anhui province of China) was 9.83 *µ*g/g, but that of Bozhou (Anhui province of China) was 32.99 *µ*g/g. Except for the place of production, time of plants collection also influences the PAs content by comparing the result of present and reported work; adonifoline content in *Senecio scandens* collected in March from Guizhou province of China was 47 *µ*g/g [14], but in present experiment adonifoline content in *Senecio scandens* collected in December also from Guizhou province of China was only 7 *µ*g/g. These data may be used to explain that why PAs contents in QT of different companies and different batches were not the same because *Senecio scandens* was the main material of QT. Another direct proof was obtained by comparing the data of the present study with reported data [14]. In *Senecio scandens* of Guangxi province of China, adonifoline was not detected, and in our experiment, the tablet of Rensheng Company also in Guangxi province contained no adonifoline.

Except for adonifoline and senkirkine identified in the present study in QT, other PAs were identified in reported data such as senecionine, seneciphylline, neoplatyphylline, and their N-oxides [8]. But these PAs were not detected in tablets. Senkirkine was another main PAs constituent in *Senecio scandens* and had a content of 2.43 *µ*g/g; but in tablets, the highest senkirkine content was 0.7 *µ*g/g. Our previews data showed that senkirkine under high temperature was not stable. According to CP, the production process of QT includes a step that extracts *Senecio scandens* with boiling water [1], so extraction temperature may be one of the reasons that some PAs could be detected in plants but not in tablets.

Although *Senecio scandens* is the legal plant source of QT, the other *Senecio* plants may be used to replace it because distinguishing dried plants from each other is very difficult when lacking an experienced botanist. So, eight *Senecio* plants were assayed to determine the PAs content to understand whether the probable replacement would influence the PAs content of tablets. Data showed that PAs contents of eight plants were various. For adonifoline, only *Senecio scandens* contained it; senkirkine existed in four plants, but its contents in *Senecio scandens* and *Tephroseris phaeantha* were high and in the other two plants, *Senecio argunensis* and *Senecio laetus*, they were low. As in QT, the quantity of PA2 in some *Senecio* plants was very low, even zero. Senecionine existed in all eight plants investigated and with high content in *Senecio vulgaris*, *Senecio cannabifolius,* and *Senecio laetus*, but in *Senecio scandens,* its content was shallow. There was no toxicity reported for adonifoline but senecionine was a received toxicant in reported data. So, if some *Senecio* plants which had high content of senecionine replaced *Senecio scandens*, the toxicity of QT may be induced. By comparing these data, we can conclude that PAs content and type in different *Senecio* species are so different that replacement use of *Senecio scandens* in QT by other *Senecio* plants must not be permitted. So, although the rule that *Senecio scandens* is the legal plant source of QT in CP is based only on traditional use in China, our data may provide some experimental support of its use.

In the present study, only limited PAs compounds were detected in QT and some *Senecio* species herbs using an HPLC/MS/MS approach in full scan mode, even according to the characteristic product ions at m/z 120/138 or 168/150 in the literature [25] and comparing with the reference standards. These may be due to the low PAs content in QT and its plant resource *S. scandens* and the variation in PAs content amongst *S. scandens* from different geographic locations [25]. On the other hand, PA2 needs to be further confirmed accurately in the future. And then, there are some other types of alkaloids in the plants of *S. scandens* except for PAs [26], though whether these types of alkaloids are toxic is unknown.

## 5. Conclusion

In conclusion, an HPLC/MS/MS method has been used in the present study to identify and determine the main PAs in QT. And these index compounds have been determined in eight *Senecio* plants. As a result, three PAs have been identified in tablets, two of them were identified as adonifoline and senkirkine, and another was presumed as emiline. Because senecionine is a common constituent in several reported *Senecio* plants and has demonstrated toxicity, senecionine content in QT and eight *Senecio* plants has also been assayed. According to our data, adonifoline was the main constituent of QT, and senecionine was not detected in any tablets. Contents of PAs were various between different manufactories and between different batches of the same producer. In eight *Senecio* plants, adonifoline was detected only in *Senecio scandens,* but senecionine was detected in all eight plants. *Senecio vulgaris*, *Senecio cannabifolius,* and *Senecio laetus* contained high level of senecionine but only a little senecionine existed in *Senecio scandens*. So as our advice, replace the use of *Senecio scandens* with other *Senecio* plants must be forbidden because senecionine had demonstrated toxicity. Plant source was considered as the main factors that influence the PAs content in QT as well as plant collection place and collection time. So, material standards must be found to control the tablet quality. Senkirkine content in QT was less than in plants because of its instability. According to these data, PAs content in some QTs was very high, but whether it can induce corresponded toxicity would need advanced pharmacology and toxicity study. Our chemical analysis data combined with advanced pharmacology and toxicology study can provide a reference for making a guideline for the safe use of QT.

## Figures and Tables

**Table 1:** Information of QTs and *Senecio* plants tested in the present study.

Company/species	Abbreviations	Batch number	Place of production
Qianbai biyan tablets	HP		
Heping Co.	GYT	50301	Guangdong province
Guoyitang Co.	HZ	20150201	Guangdong province
Huizhou Co.	BLXF	50428	Guangdong province
Boluoxianfeng Co.	BYS	50615	Guangdong province
Baiyunshan Co.	RS	L5D001	Guangdong province
Rensheng Co.	XZ	50302	Guangxi province
Xiuzheng Co.	SF	30801	Jilin province
Sifang Co.	QX	50602	Henan province
Qixing Co.	QX	5119	Guangdong province
Qixing Co.	QX	4040	Guangdong province
Qixing Co.	QX	4121	Guangdong province
Qixing Co.	QX	4126	Guangdong province
Qixing Co.	QX	5011	Guangdong province
Qixing Co.	QX	5138	Guangdong province
Qixing Co.	QX	5141	Guangdong province
Qixing Co.	QX	5159	Guangdong province
Qixing Co.	QX	6095	Guangdong province
Qixing Co.	QX	6109	Guangdong province
Qixing Co.	QX	6153	Guangdong province
Qixing Co.	QX	6168	Guangdong province
Qixing Co.	QX	6179	Guangdong province
Qixing Co.	S.sc.	6248	Guangdong province
Plant materials	S.ar.		
*S. scandens*	S.ca.v.	—	Guangdong province
*S. argunensis*	S.ca.	—	Jilin province
*S. cannabifolius* var*. integrifolius*	S.vu	—	Jilin province
*S. cannabifolius*	T.ph	—	Jilin province
*S. vulgaris*	S.la	—	Heilongjiang province
*T. phaeantha*	S.ne	—	Jilin province
*S. laetus*		—	Chongqing city
*S. nemorensis*		—	Jilin province

**Table 2:** PAs content in tablets of different companies, batches, and *Senecio* plants.

Company/species	Batch number	Adonifoline	Senecionine	Senkirkine	PA2
Heping	50301	—	—	_	—
Guoyitang	20150201	—	—	184.16	8.64
Huizhou	50428	53.04	—	65.91	—
Boluoxianfeng	50615	25.93	—	54.44	—
Baiyunshan	L5D001	799.09	—	781.40	33.44
Rensheng	50302	—	—	279.27	11.13
Xiuzheng	30801	2380.10	—	—	—
Sifang	50602	2125.64	—	—	—
Qixing	5119	10623.04	—	362.91	8.90
Qixing	4040	—	—	—	—
Qixing	4121	—	—	—	—
Qixing	4126	—	—	—	—
Qixing	5011	—	—	—	—
Qixing	5138	—	—	—	—
Qixing	5141	3088.67	—	—	—
Qixing	5159	9762.87	—	—	—
Qixing	6095	5467.22	—	—	—
Qixing	6109	15351.60	—	—	—
Qixing	6153	—	—	—	—
Qixing	6168	—	—	—	—
Qixing	6179	—	—	—	—
Qixing	6248	3449.40	—	—	—
*S. scandens*	—	7043.64	21.31	2425.01	84.85
*S. argunensis*	—	—	94.46	95.32	9.58
*S. cannabifolius* var.	—	—	17.35	—	—
*S. cannabifolius*	—	—	4125.63	—	—
*S. vulgaris*	—	—	3950.02	—	—
*T. phaeantha*	—	—	9.75	2162.57	0.00
*S. laetus*	—	—	2116.77	393.27	18.38
*S. nemorensis*	—	—	29.56	—	—

*∗*All units in the table are in ng/g.

## Data Availability

The data generated and analyzed for this study are available from the corresponding author by request.
